# A Phase II Study of Docetaxel and Epirubicin in Advanced Adult Soft Tissue Sarcomas (STS)

**DOI:** 10.1080/13577140400016705

**Published:** 2004-12

**Authors:** Haralabos P. Kalofonos, Charalabos Kourousis, Michalis V. Karamouzis, Gregoris Iconomou, Ekaterini Tsiata, Fotis Tzorzidis, Panagiotis Megas, Elias Lambiris, Vasilios Georgoulias

**Affiliations:** 1 Division of Oncology Department of Medicine University Hospital Patras Medical School Rion 26504 Greece; 2 Department of Medical Oncology School of Medicine University General Hospital of Heraclion Crete Greece; 3 Department of Orthopedics University Hospital Patras Medical School Rion Greece

## Abstract

*Purpose:* The aim of this study was to determine the efficacy and safety of docetaxel plus epirubicin combination as first-line chemotherapy in patients with locally advanced and/or metastatic adult STS.

*Patients and Methods:* Eighteen patients were treated with epirubicin 30 mg/m^2^ on days 1 to 3 and docetaxel 100 mg/m^2^ on day 1 every 3 weeks.

*Results:* Fifteen out of 18 patients (83.4%) were assessable for response. No complete response was recorded. Three (20%) patients achieved PR, 3 had SD and 9 PD. The overall median survival was 14 months (range, 3–48 months) and the median time to disease progression was 4 months (range, 2–45 months). Grade ≥ 3 neutropenia occurred in 88% and neutropenic fever in 27.8% of patients. Other toxicities were mild. No treatment related deaths occurred.

*Discussion:* Docetaxel plus epirubicin combination achieved low response rate with severe myelotoxicity in patients with advanced STS.


*Sarcoma*, December 2004, *VOL. 8, NO. 4, 129–133*

Taylor&Francis healthsciences

**ORIGINAL ARTICLE**

### A phase II study of docetaxel and epirubicin in advanced adult soft tissue sarcomas (STS)

HARALABOS P. KALOFONOS[1], CHARALABOS KOUROUSIS[2], MICHALIS V. KARAMOUZIS[1], GREGORIS ICONOMOU[1], EKATERINI TSIATA[1], FOTIS TZORZIDIS[1], PANAGIOTIS MEGAS[1], ELIAS LAMBIRIS[3] & VASILIOS GEORGOULIAS[2]

[1]*Division of Oncology, Department of Medicine University Hospital, Patras Medical School, Rion, Greece*
[2]*Department of Medical Oncology, School of Medicine, University General Hospital of Heraclion, Crete Greece*
[3]*Department of Orthopedics, University Hospital, Patras Medical School, Rion, Greece*

**Abstract**
*Purpose:* The aim of this study was to determine the efficacy and safety of docetaxel plus epirubicin combination as first-line chemotherapy in patients with locally advanced and/or metastatic adult STS.
*Patients and Methods:* Eighteen patients were treated with epirubicin 30 mg/m$^2$ on days 1 to 3 and docetaxel 100 mg/m$^2$ on day 1 every 3 weeks.
*Results:* Fifteen out of 18 patients (83.4%) were assessable for response. No complete response was recorded. Three (20%) patients achieved PR, 3 had SD and 9 PD. The overall median survival was 14 months (range, 3–48 months) and the median time to disease progression was 4 months (range, 2–45 months). Grade $\geq 3$ neutropenia occurred in 88% and neutropenic fever in 27.8% of patients. Other toxicities were mild. No treatment related deaths occurred.
*Discussion:* Docetaxel plus epirubicin combination achieved low response rate with severe myelotoxicity in patients with advanced STS.

### Introduction

Soft tissue sarcomas (STS) represent approximately 1% of all adult malignant tumors. Clinical decisions are based on a few well-recognized prognostic factors such as size, location and grading.[1] Adequate surgically local control is the treatment of choice for STS. Radical resection or limb-sparing surgery followed by radiotherapy has significantly improved local control of the disease in cases of extremity tumors.[2] However, the occurrence of distant metastases remains a common clinical problem and the main cause of death.[3]

Chemotherapy is currently being used for the treatment of locally advanced and/or metastatic STS as also in the adjuvant setting, but only a few agents have shown response rates of more than 15%. Among them the most active drugs are doxorubicin and ifosfamide as well as epirubicin, dacarbazine, dactinomycin and methotrexate.[4]

A variety of combination regimens has been studied in phase II and III trials and most of them have included adriamycin.[5–8] Most of these studies have suggested that combination chemotherapy may result in higher response rates than single-agent doxorubicin. Epirubicin, which is an analogue of doxorubicin with less cardiotoxicity but equal efficacy,[9] has been used as single agent and in combination regimens for the treatment of STS.[10–12] However, no combination regimen has been superior to single-agent adriamycin for improving survival. Possible explanations for the rather disappointing results of the adriamycin-containing regimens in advanced STS could be the relatively low dose of anthracycline and the toxicity of those combinations.

Docetaxel is a new semisynthetic compound enhancing microtubule assembly and inhibiting tubulin depolymerization, resulting in cell cycle arrest in M phase.[13] Docetaxel has been used as a second-line chemotherapy for the treatment of patients with adult advanced STS and has yielded a response rate of 17%.[14] However, in another phase II study, no responses could be detected following treatment with docetaxel although it could be related to the drug dose-intensity.[15] Furthermore, in another phase II study in previously untreated patients, docetaxel demonstrated a response rate of 11%[16],

Correspondence to: Haralabos P. Kalofonos, M.D., Ph.D., Division of Oncology, Department of Medicine, University Hospital, University of Patras Medical School, Rion 26504, Greece. Tel.: +30-2610-999535; Fax: +30-2610-994645; E-mail: kalofon@med.upatras.gr

1357-714X print/1369–1643 online © 2004 Taylor & Francis Ltd
DOI: 10.1080/13577140400016705

but in a randomized trial of docetaxel versus doxorubicin as first- and second-line chemotherapy, docetaxel failed to demonstrate any activity against STS.[17]

The aim of this study was to evaluate the efficacy and determine the safety of the docetaxel plus epirubicin combination as first-line chemotherapy in adult patients with advanced STS.

### Subjects and methods

### *Patients and eligibility requirements*

Patients aged less than 75 years, with histologically confirmed locally advanced or metastatic STS entered the study. Patients had to have at least one bidimensionally measurable lesion with evidence of progression within 6 weeks prior to treatment. Other eligibility criteria were: ECOG performance status (PS) $\leq 2$, life expectancy of > 3 months, no previous chemotherapy or radiation therapy on target lesions, no functionally important cardiovascular disease (normal left ventricular ejection fraction), adequate renal (clearance > 50 mL/min) and liver (bilirubin level < 2 mg/dl) function, adequate bone marrow reserve (absolute WBC count > 3000/ml and platelet count > 100 000/ml), absence of uncontrolled infection, no brain or leptomeningeal metastases, no second primary malignant disease and absence of pregnancy (Table 1). Informed consent was obtained from all patients according to institutional guidelines.

**Table 1.** *Patients and disease characteristics*

| | |
|---|---|
| Total number of patients enrolled | 18 |
| Assessable patients for response | 15 |
| Assessable patients for toxicity | 18 |
| Sex | |
| Male | 6 |
| Female | 12 |
| Age (years) | |
| Range | 39–73 |
| Median | 60 |
| WHO performance status | |
| 0.1 | 16 |
| 2 | 2 |
| Histology | |
| Leiomyosarcoma | 11 |
| Esophagus | 1 |
| Stomach | 3 |
| Colon | 2 |
| Uterus | 4 |
| Liver | 1 |
| Fibrosarcoma | 2 |
| Retroperitoneal sarcoma | 2 |
| Other | 3 |
| Pelvis | 1 |
| Breast | 1 |
| Sinus | 1 |
| Locally advanced | 10 |
| Metastatic | 8 |
| Lung | 6 |
| Liver | 4 |
| Bones | 3 |
| Prior therapy | |
| Chemotherapy | No |
| Radiotherapy | No |
| Surgery | 10 |

Histological subtypes were leiomyosarcoma ($n = 11$), retroperitoneal sarcoma ($n = 2$), fibrosarcoma ($n = 2$) and three of liposarcoma ($n = 1$), soft-tissue chondrosarcoma ($n = 1$) and undifferentiated sarcoma ($n = 1$). In four out of 11 patients with leiomyosarcoma, the disease was located in the uterus. None of the patients with leiomyosarcoma, after retrospective immunohistochemical evaluation of the specimens, proved to have gastrointestinal stromal tumor (GIST).

Ten patients had locally advanced STS and eight patients had metastatic disease. Sites of locally advanced disease were the pelvis (six patients), the extremities (five patients), the thoracic wall (one patient), the retroperitoneum (one patient), the abdominal cavity (two patients) and the paranasal sinus (one patient). The lung was the most frequent site of metastases (67%), followed by the liver (45%) and the bones (34%).

### *Treatment schedule and dose modifications*

Chemotherapy consisted of 30 mg/m$^2$ epirubicin on days 1–3 and 100 mg/m$^2$ docetaxel on day 1. Epirubicin (Farmorubicin; Pharmacia Italia, S.p.A., Italy) was dissolved in distilled water at a concentration of 5 mg/ml and was administered as an intravenous bolus infusion over a period of 5–20 min through saline infusion tubing. Docetaxel (Taxotere; Aventis Pharma, Bridgewater, USA) was administered as a 1 h intravenous infusion. Patients also received oral corticosteroids (methylprednisolone 32 mg, 12 and 3 h before and 12 h after docetaxel administration and 64 mg daily on days 2–4) and diphenhydramine hydrochloride. rhG-CSF (Granocyte; Aventis Pharma) (5 μg/kg per day s.c.) was administered subcutaneously prophylactically on days 4–12 in patients developing grade 3 or 4 myelosuppression after the first cycle of chemotherapy. Antiemetic therapy consisted of ondasetron (24 mg/day) for 5 days and dexamethasone whenever required. Treatment was repeated every 3 weeks for a total of six cycles. Treatment was discontinued in case of disease progression, intolerable toxicity or patient refusal.

The NCI Common Toxicity Criteria (CTC) scale was used for grading of adverse events and dose modifications. A complete blood cell count was performed twice weekly. If the neutrophil count on the day of scheduled retreatment was $< 1.5 \times 10^9$/L or the platelet count was $< 100 \times 10^9$/l, treatment was postponed for 1 week without dose adjustment. In the case of neutrophil count $< 0.5 \times 10^9$/l lasting for more than 7 days or complicated with fever, or platelet count $< 25 \times 10^9$/L, the subsequent dose of docetaxel was reduced to 75 mg/m$^2$ and the subsequent dose of epirubicin was reduced to 67.5 mg/m$^2$. In the case of left ventricular ejection fraction decrease by 10%, treatment was continued without epirubicin. In the case of grade 2 skin toxicity or neurotoxicity, a 25% reduction of the docetaxel was required. Docetaxel would be stopped in the case of persistent grade 2 skin reactions and grade 3 neurotoxicity. In the case of fluid retention, no dose reduction of docetaxel was planned, although treatment with 50 mg of spironolactone would be recommended.

### *Response criteria*

Evaluation of response was carried out according to World Health Organization scale. Initial evaluation included a thorough medical history and physical examination, complete blood count with differential, biochemistry profile and clotting studies. Pretreatment imaging evaluation included: chest X-ray, computed tomography (CT) scans of the lungs and abdomen, whole body ultrasound, bone scintigraphy, ECG and echocardiogram or MUGA. Additional CT scan or MRIs were performed if clinically indicated. During treatment, clinical and hematology laboratory evaluation were performed every 3 weeks. Imaging studies were made at baseline, after cycle 3 and at the end of chemotherapy or even earlier if there was clinical or other evidence of relapse.

### *Statistical analysis*

Time to event curves was estimated using the Kaplan–Meier method. Survival was calculated from the date of registration to the date of death, irrespective of its cause. The duration of partial response or stable disease was calculated from the first documentation of response until the date of radiologically documented progression. Time to disease progression was determined by the interval between the initiation of therapy to the first date that disease progression was objectively documented.

The study followed Simon's two-stage optimal design. A response rate of more than 20% was the cut-off for considering the schedule sufficiently active. The first step consisted of 18 patients. If more than three responses were observed, accrual was to continue to a total of 35 patients with a 5% rejection error and a power of 90%.

### Results

### *Patient demographics*

Between January 1997 and April 2001, 18 chemotherapy- and radiation-naïve patients with locally advanced or metastatic soft tissue sarcomas consented to participate in the study (Table 1). Their median age was 60 years and 16 of them had an ECOG PS of 0-1. Eleven (61%) patients had leiomysarcoma. Additionally, 10 (55.5%) patients had locally advanced and eight (44.4%) metastatic disease.

### *Drug delivery*

A total of 70 chemotherapy courses were administered with a median number of four cycles/patient (range 1–6). Six (33.3%) patients only completed six cycles of the treatment. The median delivered dose intensity was 39.6 mg/m$^2$/week for docetaxel and 35.7 mg/m$^2$/week for epirubicin, corresponding to 99 and 99% of the protocol-planned doses, respectively.

### *Efficacy of treatment*

In an intention-to-treat analysis, no complete response was observed, while partial response was achieved in three patients (20%, 95%CI:3.0–43%). Partial responses were observed in a patient with soft-tissue chondrosarcoma, a patient with uterus leiomyosarcoma and another with undifferentiated sarcoma. Three patients (20%, 95%CI:3.0–43%) had stable disease and nine (60%, 95%CI: 32–88%) progressive disease. The median time to disease progression was 4 months (range, 2–45 months), and the overall median survival of all patients was 14 months (range, 3–48 months) (Fig. 1). The 1-year survival was 73% (95%CI:48–99%). The median time to disease progression for the three responding patients was 12 months (range, 8–45 months) and the median overall survival 26 months (range, 4–48 months).

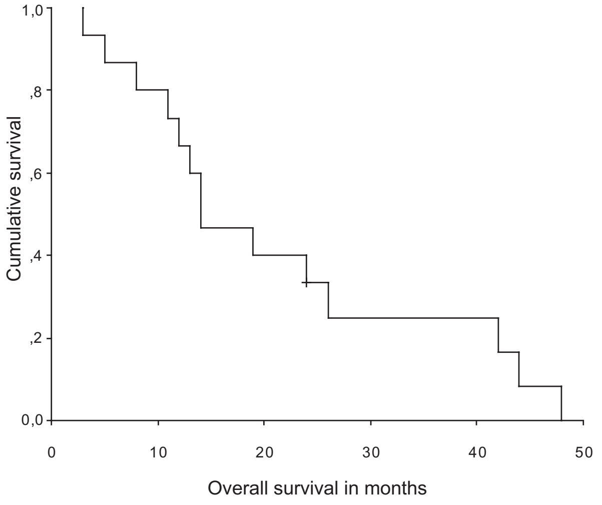

**Fig. 1.** *Overall survival of the patients enrolled in the study.*

**Table 2.** *Toxicity profile*

| Toxicity | Grade 1 | Grade 2 | Grade 3 | Grade 4 |
|---|---|---|---|---|
| Neutropenia | 0 | 2 | 8 | 8 |
| Febrile neutropenia | 0 | 0 | 5 | 0 |
| Anemia | 6 | 2 | 0 | 0 |
| Alopecia | 3 | 6 | 9 | 0 |
| Nausea/vomiting | 3 | 2 | 1 | 0 |
| Fatigue | 6 | 3 | 0 | 0 |
| Stomatitis | 2 | 0 | 0 | 0 |
| Constipation | 2 | 1 | 0 | 0 |
| Diarrhea | 1 | 0 | 0 | 0 |
| Neuromotor side effects | 0 | 1 | 0 | 0 |
| Neurosensory side effects | 5 | 1 | 0 | 0 |
| Fluid retention syndrome | 1 | 0 | 0 | 0 |

*Toxicity and complications*

All patients were assessed for toxicity. No toxic death occurred. The major adverse reaction was hematological toxicity (Table 2). Neutropenia was recorded in all patients; grade 2 in two (11.1%) patients, grade 3 in eight (44.4%) patients and grade 4 in eight (44.4%) patients. Neutropenic fever that required administration of antibiotics, was reported in five (27.8%) patients. Anemia was mild; six (33.3%) patients experienced grade 1 and two (11.1%) patients grade 2. There was no case of thrombocytopenia. In three (16.7%) patients, subsequent dose reduction of the chemotherapeutic agents was required due to prolonged myelotoxicity.

Grade 1 neurotoxicity was recorded in five (27.8%) patients; one patient presented grade 2 neurosensory defects and neuromotor symptoms and required reduction of docetaxel dose. Treatment-related nausea and vomiting was reported in six (33.3%) patients, but only one patient reported grade 3 toxicity requiring the addition of dexamethasone. Furthermore, two (11.1%) patients demonstrated grade 1 stomatitis, one (5.5%) patient grade 1 diarrhea, and three (16.7%) patients complained of mild constipation. Alopecia grade 3 related to chemotherapy was observed in nine (50%) patients. No patient demonstrated skin reactions. One patient presented fluid retention syndrome with edema and pleural effusion with no delay in the treatment schedule.

### Discussion

The aim of this study was to evaluate the clinical efficacy and toxicity of the docetaxel and epirubicin combination as first-line chemotherapy in advanced adult soft tissue sarcomas. Initial phase II studies involving patients with advanced STS have demonstrated response rates on docetaxel treatment of 11 and 17%.[14,16] The rationale for the combination of docetaxel and epirubicin is based on their activity against STS, their limited cross-resistance as well as the different but potentially synergistic mechanisms of action, and the promising results of the combination treatment in other malignancies such as breast cancer.[18] Both anthracyclines and taxanes are known to have a dose–response relationship and some overlapping toxicities, so the combined use of both agents results in an enhanced toxicity profile. We chose the 3-day schedule of epirubicin in order to achieve better clinical outcome and reduce dose-dependent toxicities.

This trial could not meet the predefined efficacy criteria. A possible explanation of the low response rate observed following the administration of docetaxel together with epirubicin may be partly attributed to the limited activity of docetaxel in STS and the high percentage of patients enrolled with leiomyosarcomas of retroperitoneal and visceral origin (61.1%),[19] while only 4 patients (22%) had uterine leiomyosarcomas. The limited activity of docetaxel as first-line chemotherapy agent in advanced STS was also outlined in a randomized study comparing docetaxel versus doxorubicin with optimal dose-intensity.[17] However, docetaxel seems to represent an efficacious and tolerable treatment in patients with uterine leiomyosarcomas in combination with gemcitabine.[20]

Another limitation of our study was the considerable myelosuppression associated with the chemotherapy regimen, although no toxic death occurred. Indeed, neutropenia was the immediate dose-limiting toxicity. Neutropenia appeared in almost all patients and it was grades 3 and 4 in 16 of the patients (88.9%). Neutropenic fever was observed in five (27.8%) patients, and in three (16.7%) patients the dose was reduced because of prolonged myelotoxicity. Docetaxel was administered at the maximum accepted dose according to pharmacokinetic studies.[21,22] Therefore, this study seems to provide the best results that can be obtained in terms of dose–response effect. In addition, dose escalation of epirubicin could possibly increase the risk of cardiotoxicity. Fluid retention syndrome represents a common toxicity of docetaxel, but it was demonstrated in only one patient while hypersensitivity reactions did not occur. The prophylactic use of corticosteroids might explain the low incidence of these adverse reactions. The majority of patients did not experience other significant toxicities, except in one patient with a grade 2 neurosensory and neuromotor adverse event who required reduction of docetaxel dose.

To summarize, the present study demonstrates that the combination of docetaxel with epirubicin for the treatment of patients with advanced STS has considerable myelotoxicity and rather low efficacy, and should not be used in adult soft tissue sarcomas.

### References

1. Guillou L, Coindre JM, Bonichon F, Nguyen BB, Terrier P, Collin F, Vilain MO, Mandard AM, Le Doussal V, Leroux A, Jacquemier J, Duplay H, Sastre-Garau X, Costa J. Comparative study of the National Cancer Institute and French Federation of Cancer Centers Sarcoma Group grading systems in a population of 410 adult patients with soft tissue sarcoma. *J Clin Oncol* 1997; 15: 350–62.
2. Moley JF, Eberlein TJ. Soft-tissue sarcomas. *Surg Clin North Am* 2000; 80: 687–708.
3. Fong Y, Coit DG, Woodruff JM, Brennan MF. Lymph node metastasis from soft tissue sarcomas in adults: analysis of data from a prospective database of 1772 sarcoma patients. *Ann Surg* 1993; 217: 72–7.
4. Patel SR. Recent advances in systemic therapy of soft tissue sarcomas. *Exp Rev Anticancer Ther* 2003; 3: 179–84.
5. Yap BS, Baker LH, Sinkovics JG, Rivkin SE, Bottomley R, Thigpen T, Burgess MA, Benjamin RS, Bodey GP. Cyclophosphamide, vincristine, adriamycin and DTIC (CYVADIC) combination chemotherapy for the treatment of advanced sarcomas. *Cancer Treat Rep* 1980; 64: 93–8.
6. Elias A, Ryan L, Sulkes A, Collins J, Aisner J, Antman KH. Response to mesna, doxorubicin, ifosfamide and dacarbazine in 108 patients with metastatic or unresectable sarcoma and no prior chemotherapy. *J Clin Oncol* 1989; 7: 1208–16.
7. Edmonson JH, Long HJ, Richardson RL. Phase II study of a combination of mitomycin, doxorubicin and cis-platin in advanced sarcomas. *Cancer Chemother Pharmacol* 1985; 15: 181–2.
8. Kalofonos HP, Bafaloukos D, Kourelis TG, Karamouzis MV, Megas P, Iconomou G, Tsiata E, Dimitropoulos D, Kosmidis P, Lampiris E. Adriamycin and cis-platimun as first-line treatment in unresectable locally advanced or metastatic adult soft-tissue sarcomas. *Am J Clin Oncol* 2004; 27: 307–11.
9. Launchbury AP, Habbouri N. Epirubicin and doxorubicin: a comparison of their characteristics, therapeutic activity and toxicity. *Cancer Treat Rev* 1993; 19: 197–228.
10. Reichardt P, Tilgner J, Hohenberger P, Dorken B. Dose-intensive chemotherapy with ifosfamide, epirubicin, and filgrastim for adult patients with metastatic or locally advanced soft tissue sarcoma: a phase II study. *J Clin Oncol* 1998; 16: 1438–43.
11. Palumbo R, Neumaier C, Cosso M, Bertero G, Raffo P, Spadini N, Valente S, Villani G, Pastorino M, Toma S. Dose-intensive first-line chemotherapy with epirubicin and continuous infusion ifosfamide in adult patients with advanced soft tissue sarcomas: a phase II study. *Eur J Cancer* 1999; 35: 66–72.
12. Nielsen OS, Dombernowsky O, Mouridsen H, Crowther D, Verwij J, Buesa J, Steward W, Daugaard S, van Glabbeke M, Kirkpatrick A, Tursz T. High-dose epirubicin is not an alternative to standard-dose doxorubicin in the treatment of advanced soft tissue sarcomas. A study of the EORTC soft tissue and bone sarcoma group. *Br J Cancer* 1998; 78: 1634–49.
13. Eahart RH. Docetaxel (Taxotere): Preclinical and general clinical information. *Semin Oncol* 1999; 26: 8–13.
14. van Hoesel GCM, Verweij G, Catimel G, Clavel M, Kerbat P, van Oosterom T, Kerger J, Tursz T, van Glabbeke M, van Pottelsberghe. Phase II study with Docetaxel (Taxotere) in advanced soft tissue sarcomas of the adult. *Ann Oncol* 1994; 5: 539–42.
15. Edmonson JH, Ebbert LP, Nascimento AG, Jung SH, McGaw H, Gerstner JB. Phase II study of docetaxel in advanced soft tissue sarcomas. *Am J Clin Oncol* 1996; 19: 574–6.
16. Bramwell V, Blackstein M, Berlanger K. A phase II study of docetaxel in chemotherapy-naïve patients with recurrent or metastatic adult soft tissue sarcoma. *Oncology* 1998; 2: 29–33.
17. Verweij J, Lee SM, Ruka W, Buesa J, Coleman R, van Hoessel R, Seynaeve C, Donato di Paola E, van Glabbeke M, Tonelli D, Judson IR. Randomized phase II study of docetaxel versus doxorubicin in first- and second-line chemotherapy for locally advanced or metastatic soft tissue sarcomas in adults: A study of the European Organization for Research and Treatment of Cancer Soft Tissue and Bone Sarcoma group. *J Clin Oncol* 2000; 18: 2081–6.
18. Nabholtz JMC, Riva A. Taxane/anthracycline combinations: Setting a new standard in breast cancer? *Oncologist* 2001; 6 (Suppl 3): 5–12.
19. van Glabbeke M, van Oosterom AT, Oosterhuis JW, Mouridsen H, Crowther D, Somers R, Verweij J, Santoro A, Buesa J, Tursz T. Prognostic factors for the outcome of chemotherapy in advanced soft-tissue sarcoma: an analysis of 2,185 patients treated with anthracycline-containing first-line regimens – A European Organization for Research and Treatment of Cancer, Soft Tissue and Bone Sarcoma Group Study. *J Clin Oncol* 1999; 17: 150–7.
20. Hensley ML, Maki R, Venkatraman E, Geller G, Lovegren M, Aghajanian C, Sabbatini P, Tong W, Barakat R, Spriggs DR. Gemcitabine and docetaxel in patients with unresectable leiomyosarcoma: results of a phase II trial. *J Clin Oncol* 2002; 20: 2824–31.
21. Airoldi M, Cattel L, Pedani F, Marchionatti S, Tagini V, Bumma C, Recalenda V. Clinical and pharmacokinetic data of a docetaxel-epirubicin combination in metastatic breast cancer. *Breast Cancer Res Treat* 2001; 70: 185–95.
22. Hirth J, Watkins PB, Strawderman M, Schott A, Bruno R, Baker LH. The effect of an individual's cytochrome CYP3A4 activity on docetaxel clearance. *Clin Cancer Res* 2000; 6: 1255–8.
